# Basal cell carcinoma of the head and neck: Surgical management and postoperative strategies – A researcher’s perspective

**DOI:** 10.18632/oncoscience.646

**Published:** 2026-03-11

**Authors:** Lawik Revend, Doha Revend, Florian Dudde

**Affiliations:** ^1^Department of Orthopedic and Trauma Surgery, Division of Plastic Surgery, Army Hospital Berlin, Berlin, Germany; ^2^Department of Otolaryngology – Head and Neck Surgery, Army Hospital Berlin, Berlin, Germany; ^3^Department of Oral and Maxillofacial Surgery, Army Hospital Hamburg, Hamburg, Germany

**Keywords:** basal cell carcinoma, surgical excision, local flap, head and neck reconstruction, plastic surgery

## Abstract

Introduction: Basal cell carcinoma (BCC) is one of the most common cutaneous malignancies and predominantly affects sun-exposed regions, particularly the head and neck. Although metastatic spread is rare, locally invasive growth may compromise functionally and aesthetically critical structures. Effective management therefore requires not only oncologic tumor control but also carefully planned reconstruction and long-term follow-up.

Materials and Methods: This study presents a structured narrative review of the literature combined with institutional clinical experience. Relevant publications on diagnosis, surgical management, reconstructive techniques, and postoperative strategies for head and neck BCC were identified through searches of PubMed, Embase, and the Cochrane Library. Clinical studies, systematic reviews, and guidelines published between 2000 and 2025 were analyzed and synthesized narratively due to heterogeneity in study designs and outcome measures.

Results: Surgical excision remains the cornerstone of curative treatment, with recommended margins depending on tumor risk stratification. Mohs micrographic surgery provides superior margin control in anatomically critical regions. Reconstruction must be individualized based on defect size, anatomical subunit involvement, and patient factors. Local flaps are commonly used for small- to medium-sized defects, whereas larger or deeply infiltrative tumors may require regional or free tissue transfer.

Discussion: Successful management of head and neck BCC requires interdisciplinary collaboration and individualized reconstructive planning. Advances in flap techniques, digital surgical planning, and systemic therapies have expanded treatment possibilities, enabling improved functional and aesthetic outcomes in complex cases.

## INTRODUCTION

Basal cell carcinoma (BCC) represents the most prevalent cutaneous malignancy in fair-skinned populations, accounting for nearly 80% of non-melanoma skin cancers. Its clinical and surgical significance lies in its predilection for chronically sun-exposed areas, particularly the head and neck region, where both aesthetic and functional structures are at risk [1, 2]. The global burden of BCC is rising significantly, both in terms of patient numbers and healthcare costs, due to increasing longevity and cumulative UV exposure. Although BCC rarely metastasizes, its potential for local invasion mandates timely surgical management. Plastic surgeons, dermatologic surgeons, and maxillofacial teams are increasingly challenged by the rising incidence of facial BCC, which now accounts for nearly 60 % of facial non-melanoma skin cancers requiring reconstruction [1, 3–5]. This perspective synthesizes current best practices for surgical and postoperative management of BCC of the head and neck, with particular focus on reconstructive decision-making and interdisciplinary treatment strategies. In addition to the literature review, this manuscript incorporates real-world observations from our clinical environment. Given the referral patterns and the complexity of facial skin cancer cases treated in our surgical department, the unit functions operationally as a tertiary-level reconstructive service within the German military medical system. These institutional insights are included to illustrate decision pathways in areas where published evidence remains limited and to strengthen the intended “researcher’s perspective” character of this work.

## METHODS

This manuscript was developed as a structured narrative review supported by institutional experience. Literature searches were conducted in PubMed, Embase, and the Cochrane Library between October 2024 and January 2025 using combinations of the following keywords: “basal cell carcinoma,” “head and neck,” “Mohs surgery,” “local flap,” “reconstruction,” and “systemic therapy.” Studies were eligible if they focused on basal cell carcinoma of the head and neck and reported diagnostic, surgical, or reconstructive outcomes. Clinical studies, systematic reviews, and evidence-based guidelines published between 2000 and 2025 were considered for inclusion. Non-English publications, duplicated datasets, and studies without surgical or postoperative relevance were excluded.

Due to heterogeneity in study designs, patient characteristics, and outcome metrics, quantitative synthesis or meta-analysis was not feasible. Instead, relevant findings were synthesized narratively with an emphasis on clinical applicability. Institutional surgical experience from a tertiary referral center was incorporated to contextualize decision-making in areas where high-level evidence remains limited. As a narrative review, this methodology carries inherent limitations, including potential selection bias and variability in evidence quality.

## CLINICAL PRESENTATION

Patients with BCC typically present with slowly enlarging, often asymptomatic lesions, varying morphologically by subtype: nodular, superficial, sclerosing/morpheaform, pigmented, or ulcerative (ulcus rodens) [6, 7]. Head and neck involvement is most frequent, with the nasal unit (31.8 %), periorbital region (13.6 %), and cervical zone (12.5 %) being commonly affected [1, 7]. Clinical vigilance is essential, especially in elderly or immunosuppressed patients [8]. Neglected tumors may ulcerate or infiltrate deeper structures, including musculature and cartilage, complicating surgical excision and reconstruction [5, 9].

## DIAGNOSTIC STRATEGIES

**Figure 1:**
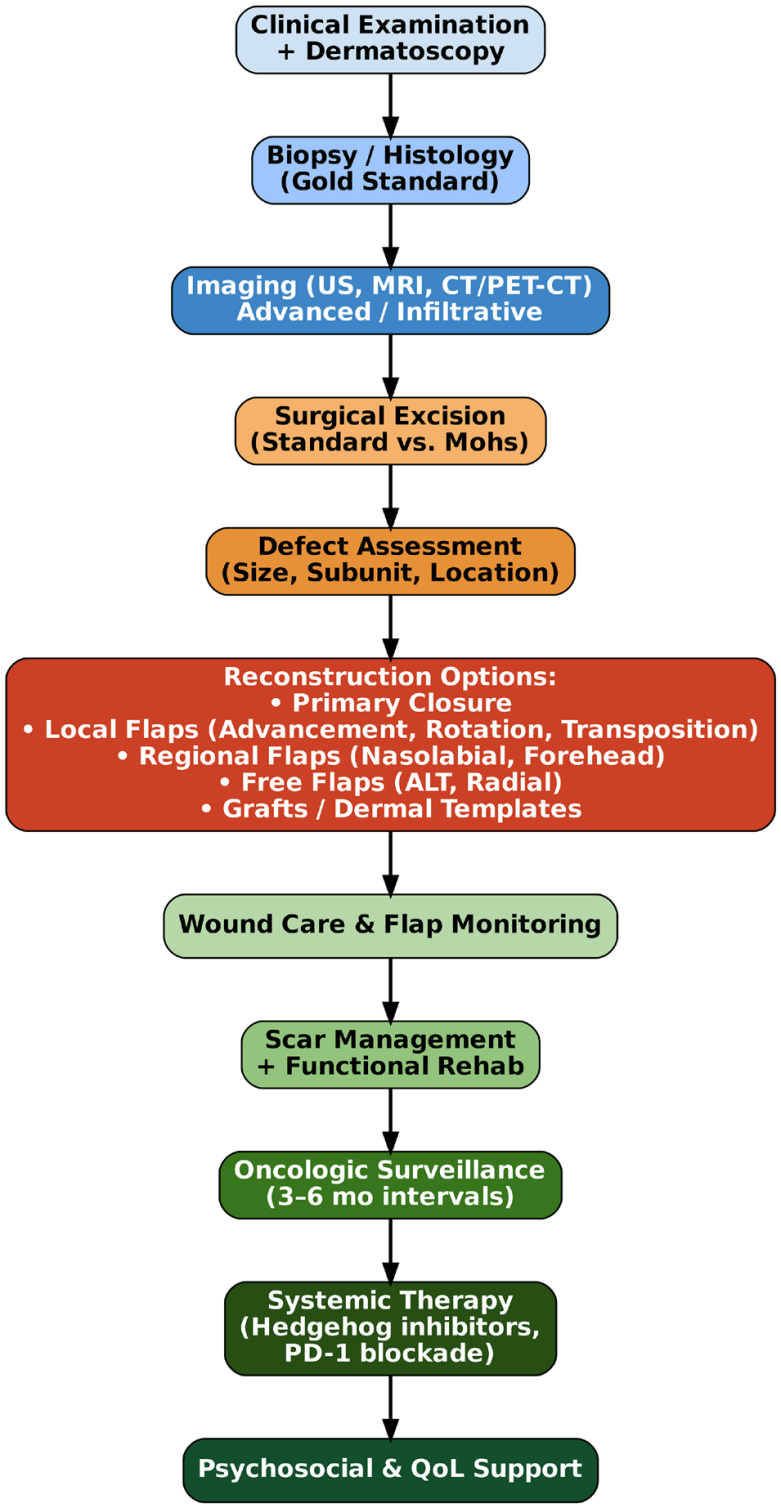
Diagnostic workflow for basal cell carcinoma of the head and neck.

The initial diagnostic evaluation involves a comprehensive full-body skin examination combined with dermoscopic assessment. Histopathological confirmation through biopsy remains the gold standard for definitive diagnosis [10, 11]. High-resolution ultrasound or MRI may be indicated to delineate tumor depth and involvement of critical structures such as the periorbital musculature or nasal cartilage [6, 7, 10] (Figure 1).

Histological confirmation, especially for aggressive variants (e.g., basosquamous, morpheaform), is essential for planning margins and reconstruction. In extensive or infiltrative disease, preoperative imaging with CT or PET/CT may be necessary to assess for bone erosion or deep soft tissue infiltration [5, 10, 11].

## SURGICAL MANAGEMENT

Surgical excision remains the cornerstone of curative BCC treatment. Recommended margins vary by risk stratification, from 3 mm in low-risk lesions to 10 mm in high-risk tumors [10]. However, these recommendations are largely derived from expert consensus statements and retrospective studies; prospective randomized evidence defining optimal margin width remains limited. Conventional excision remains standard, while Mohs micrographic surgery (MMS) offers superior margin control in functionally sensitive zones [10, 12].

Recent prospective evaluations have shown that immediate reconstruction following tumor excision - especially in nasal BCCs - does not increase recurrence risk and improves patient-reported outcomes [13]. Particularly in central facial areas, the forehead flap has emerged as a single-modality treatment offering both reliable vascularity and favorable cosmetic outcomes [14]. In a 50-patient cohort, no recurrences and high functional preservation were noted using this technique [4]. This method is ideally suited for large nasal, medial cheek, and glabellar defects.

### Reconstructive techniques

**Figure 2:**
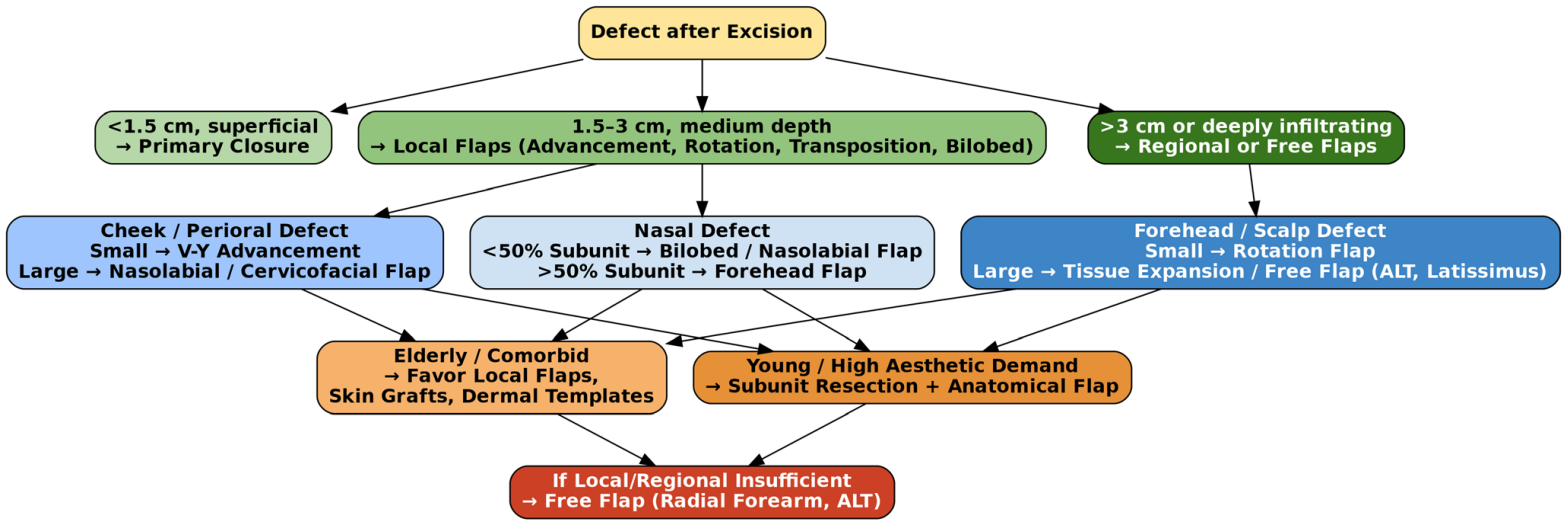
Institutional algorithm for reconstructive decision-making following excision of basal cell carcinoma in the head and neck region.

The reconstruction of surgical defects following BCC excision in the head and neck region represents a core component of functional and aesthetic patient outcomes [3, 5, 14] (Figure 2). Figure 2 provides a structured algorithm guiding flap selection based on defect size, anatomical subunit reconstruction principles, and patient comorbidities. Notably, practice patterns vary significantly—particularly in perinasal and medial cheek defects—where surgeon experience, tissue laxity, and patient-related factors influence the decision between nasolabial, bilobed, advancement, or paramedian forehead flaps. Variability in surgical preference reflects both anatomical complexity and heterogeneity in available comparative evidence. Given the anatomical complexity and cosmetic importance of the face, reconstructive planning must be individualized, taking into account defect location, size, depth, skin laxity, and patient comorbidities [14, 15]. A widely adopted principle in modern facial reconstruction is the subunit-based approach. This concept dictates that when more than 50% of a recognized aesthetic subunit (such as the nasal tip, alar lobule, or upper lip) is lost, the entire subunit should be excised and reconstructed to maintain harmonious contours and prevent patchwork deformity [15, 16]. Such strategic resection allows for the use of well-matched adjacent tissue, ultimately improving aesthetic integration. Local flap techniques are the mainstay for most small- to medium-sized defects due to their superior match in skin tone, texture, and thickness [16, 17]. These include advancement flaps for linear extension of adjacent tissue, rotation flaps to pivot skin around a defect, and transposition flaps such as bilobed and rhomboid flaps for defects of the nose, cheek, and temple. Island flaps and perforator-based designs (e.g., V-Y advancement flaps) are especially valuable in perioral and perinasal areas, allowing for mobility with minimal donor-site morbidity [15, 17]. In elderly patients, particularly those with limited healing capacity or extensive sun-damaged skin, dermal regeneration templates followed by delayed split-thickness grafting can provide durable and cosmetically acceptable results, especially for concave surfaces or large scalp defects [15, 17].

For larger or deeply infiltrative tumors, such as giant basal cell carcinomas involving the cheek, forehead, or scalp, the reconstructive challenge intensifies [14, 17]. Here, the use of tissue expansion can allow for primary closure with native skin after tumor clearance. In more extensive cases, free flap reconstruction—such as radial forearm or anterolateral thigh (ALT) flaps - is necessary to restore contour, volume, and skin coverage. Preoperative virtual planning with 3D modeling has emerged as a valuable adjunct to guide flap selection, contouring, and symmetry, especially when bilateral or multi-subunit defects are involved [8, 17, 18]. In all cases, the goal is to restore both appearance and function while minimizing donor site morbidity. Mastery of a broad reconstructive repertoire, along with attention to subunit principles, enables the surgical team to tailor outcomes to individual patient anatomy and expectations.

## POSTOPERATIVE MANAGEMENT

Effective postoperative management following surgical excision of BCC is critical to ensure wound healing, functional preservation, aesthetic outcome, and early detection of recurrence. Management strategies must be tailored to the surgical technique used, patient comorbidities, and tumor characteristics.

### Wound care and early recovery

Immediately post-surgery, wound care protocols are based on the reconstructive modality [14, 19]. For primary closures and local flaps, the use of non-adherent dressings and scheduled suture removal (typically 5–7 days for facial areas) is standard [19]. For skin grafts, bolstering techniques are applied to secure graft adherence, and monitoring for hematoma or graft necrosis is essential during the first 48–72 hours. Topical antibiotic ointments and sterile coverage are used routinely until epithelialization is complete [14, 15, 17]. In patients who undergo complex reconstructions, such as regional or free flaps, monitoring flap viability is a central aspect. Parameters such as color, capillary refill, temperature, and doppler signals are evaluated at regular intervals during the first postoperative week. In high-risk flaps, hospital admission may be necessary for observation [10, 15, 17]. Pain management should be individualized and adjusted according to defect size and surgical extent. Non-opioid analgesics such as acetaminophen or NSAIDs are often sufficient, though deeper reconstructions may require short-term opioid use [19, 20].

### Scar management and functional rehabilitation

Once wounds are stable, the emphasis shifts toward scar modulation [19]. Silicone gel sheeting, massage therapy, and sun protection are initiated approximately two weeks postoperatively [15, 19]. In hypertrophic or cosmetically significant scars, intralesional corticosteroids or laser therapy may be employed [21]. Contracture prevention, especially near the perioral or periocular region, involves early mobilization, physical therapy, and in some cases, revision surgery [15, 21]. Patients with flaps involving dynamic facial units may benefit from targeted physiotherapy to retrain mimic musculature and minimize asymmetry [15, 16]. In elderly patients or those with neuromuscular deficits, functional recovery may require more time and interprofessional input.

### Oncologic surveillance

Oncological follow-up is based on risk stratification. High-risk patients - those with aggressive histology, perineural invasion, incomplete excision, or recurrent tumors—require more intensive surveillance. Clinical examination is advised every 3–6 months in the first two years, then every 6–12 months up to five years [10, 12].

Dermoscopy can aid in early detection of subtle recurrences. In select patients, especially those with extensive prior BCC history, digital total-body photography and serial imaging may be warranted [7].

For patients treated with adjuvant radiotherapy, late effects such as fibrosis, telangiectasia, and radiation dermatitis must be monitored [11, 22]. Coordination with radiation oncology is key, particularly in the assessment of irradiated flap viability and secondary tumor development [11].

### Integration of systemic therapy

Systemic therapies are particularly relevant for patients with advanced, recurrent, or inoperable BCC. Hedgehog pathway inhibitors (vismodegib, sonidegib) have been approved for locally advanced or metastatic BCC, showing response rates between 43% and 60% in pivotal trials [23, 24]. Common adverse effects include muscle cramps, alopecia, and dysgeusia. In tumors refractory to these agents, the PD-1 inhibitor cemiplimab offers an additional option with response rates near 30% in early-phase trials [25, 26]. Importantly, neoadjuvant use of systemic therapies has enabled surgical downstaging in selected patients, allowing for function-preserving resections. Integration into surgical planning requires close collaboration between surgical oncology and dermatology/oncology. Neoadjuvant systemic therapy is considered when tumor size or anatomical location would otherwise necessitate multi-stage or high-morbidity reconstruction. Reduction in tumor volume may allow for single-stage closure or avoidance of free-tissue transfer.

### Psychosocial and quality-of-life aspects

BCC treatment, particularly involving the face, may have profound psychological and social implications. Early referral to psycho-oncologic services is advisable for patients exhibiting distress, body image disturbance, or depression. Patient-reported outcome tools, such as the FACE-Q Skin Cancer Module, can support tailored postoperative counseling and guide future reconstructive interventions [13, 27].

In summary, postoperative management is a dynamic, multidisciplinary effort that extends beyond wound closure. It encompasses structured follow-up, scar care, functional training, and holistic patient support to achieve optimal long-term outcomes. Successful treatment of facial BCC often depends on interdisciplinary collaboration among dermatologists, oncologists, pathologists, plastic surgeons, and radiation oncologists. For patients with advanced, recurrent, or inoperable BCC, systemic therapies like Hedgehog inhibitors (vismodegib, sonidegib) and emerging PD-1 inhibitors provide new therapeutic avenues [6, 10].

Plastic surgeons must be aware of the dermatologic and oncologic implications of systemic therapy, as neoadjuvant strategies can downsize tumors and allow for less morbid resections. In elderly patients with multiple comorbidities, a conservative, coordinated approach often yields better outcomes than aggressive excision alone [10, 14].

## CRITICAL REFLECTION ON LITERATURE

While summarizing clinical best practices, we now highlight the level of evidence in key sections. For example, the superiority of Mohs surgery in margin control is supported by comparative cohort studies, but prospective trials are lacking. Similarly, flap selection relies heavily on retrospective case series and institutional experience, underscoring the need for higher-level evidence. In addition, complication rates and aesthetic outcomes reported in current flap comparisons vary by reporting standards and surgeon expertise. This limits the generalizability of conclusions.

### Institutional experience and clinical perspective

In our institutional practice at the Bundeswehrkrankenhaus Hamburg, a tertiary referral center for complex facial reconstruction, basal cell carcinoma cases account for a significant portion of head and neck oncologic surgeries. Based on our experience, flap selection is often driven not only by defect size and location but also by patient-specific factors such as comorbidities, anticoagulation status, and the anticipated compliance with postoperative care. For elderly patients with perinasal or medial cheek defects, we routinely favor the nasolabial flap due to its low donor-site morbidity and superior aesthetic integration. In contrast, the forehead flap remains our technique of choice for midline nasal defects requiring robust vascular supply and subunit restoration. Notably, in the past two years we have increasingly incorporated virtual planning for flap dimensioning and symmetry control, particularly in patients with prior surgeries or scarred facial tissue. We have also observed a growing subset of patients with locally advanced or previously neglected BCC, often due to delayed access or underestimation of the lesion’s extent. In these cases, a combined approach involving neoadjuvant systemic therapy followed by staged resection and reconstruction has shown promise in reducing surgical morbidity while maintaining oncologic safety.

This evolving experience underscores the need for individualized strategies that go beyond textbook algorithms and highlights the value of interdisciplinary collaboration, especially with dermatology and oncology teams, to optimize both function and form in facial reconstruction.

## CONCLUSIONS

Basal cell carcinoma of the head and neck poses complex challenges that extend beyond simple tumor excision. Achieving successful outcomes requires not only surgical precision but also an in-depth understanding of facial aesthetics, reconstructive anatomy, and interdisciplinary collaboration. While excision remains the cornerstone of treatment, the growing diversity and complexity of cases underscore the need for adaptable reconstructive strategies, long-term surveillance, and patient-centered decision-making. Contemporary facial reconstruction increasingly draws upon subunit-based planning, novel flap techniques, and digital planning tools to achieve optimal oncologic and aesthetic results. Surgeons are now expected to master a wide array of reconstructive options and to tailor their choices based on individual patient factors such as age, comorbidity profile, tissue quality, and personal expectations. Ongoing innovation in flap design and integration of neoadjuvant systemic therapies continue to redefine what is surgically achievable.

This perspective contributes distinct value by blending structured reconstructive algorithms with institution-based insights and practical reasoning. Rather than reiterating existing guidelines, our work illustrates how reconstruction is adapted in clinical reality - especially in complex cases involving elderly, frail, or comorbid patients, where rigid protocols may not apply. By emphasizing both anatomical fidelity and functional restoration, and by providing visual schematics to guide flap selection, this manuscript offers a bridge between conceptual knowledge and applied surgical judgment. To further strengthen the evidence base, future prospective studies comparing long-term reconstructive outcomes—especially across different flap types and patient risk profiles—are essential. Moreover, expanding the role of 3D planning and evaluating quality-of-life metrics will be critical in advancing patient-centered care in facial skin cancer surgery.
